# A computer-aided diagnosis system for detecting various diabetic retinopathy grades based on a hybrid deep learning technique

**DOI:** 10.1007/s11517-022-02564-6

**Published:** 2022-05-11

**Authors:** Eman AbdelMaksoud, Sherif Barakat, Mohammed Elmogy

**Affiliations:** Faculty of Computers and Information, Mansoura University, Mansoura, P.O. 35516, Egypt

**Keywords:** Diabetic retinopathy (DR), Convolution neural network (CNN), Transfer learning, EyeNet, DenseNet, E-DenseNet

## Abstract

**Abstract:**

Diabetic retinopathy (DR) is a serious disease that may cause vision loss unawares without any alarm. Therefore, it is essential to scan and audit the DR progress continuously. In this respect, deep learning techniques achieved great success in medical image analysis. Deep convolution neural network (CNN) architectures are widely used in multi-label (ML) classification. It helps in diagnosing normal and various DR grades: mild, moderate, and severe non-proliferative DR (NPDR) and proliferative DR (PDR). DR grades are formulated by appearing multiple DR lesions simultaneously on the color retinal fundus images. Many lesion types have various features that are difficult to segment and distinguished by utilizing conventional and hand-crafted methods. Therefore, the practical solution is to utilize an effective CNN model. In this paper, we present a novel hybrid, deep learning technique, which is called E-DenseNet. We integrated EyeNet and DenseNet models based on transfer learning. We customized the traditional EyeNet by inserting the dense blocks and optimized the resulting hybrid E-DensNet model’s hyperparameters. The proposed system based on the E-DenseNet model can accurately diagnose healthy and different DR grades from various small and large ML color fundus images. We trained and tested our model on four different datasets that were published from 2006 to 2019. The proposed system achieved an average accuracy (ACC), sensitivity (SEN), specificity (SPE), Dice similarity coefficient (DSC), the quadratic Kappa score (QKS), and the calculation time (T) in minutes (m) equal $91.2\%$, $96\%$, $69\%$, $92.45\%$, 0.883, and 3.5*m* respectively. The experiments show promising results as compared with other systems.

**Graphical abstract:**

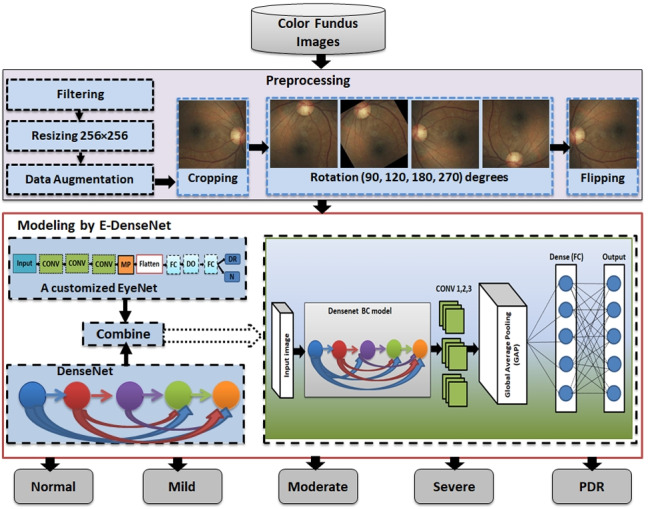

## Introduction

The human eye anatomy includes iris, cornea, pupil, lens, vitreous, macula, retina, and optic nerve. The cornea is the front of the eye and transfers light to it. Iris and its dark aperture pupil regulate the amount of entered light. The lens is the transparent structure that converges the light rays on the retina. The retina is the light-sensitive tissue of the eye’s back surface. It creates electrical impulses that cross through the optic nerve (ON) to the brain. Therefore, we can define ON as the connection between the eye and the brain’s visual cortex. Vitreous fills the middle of the eye [1]. Following the previous anatomy, the retina includes the macula, optic disc (OD), blood vessels (arteries and veins) (BV), and fovea. The macula is a small area in the retina that surrounds the fovea and includes special light-sensitive cells. These cells give the human the capability to see the details clearly. From the retina’s importance in the human eye, we concentrated on the most famous disease, which is diabetic retinopathy (DR). DR is the most complication of diabetes that is resulting from the elevation of the glucose in the blood. DR may damage the retina and cause blindness suddenly. It is a progressive disease that needs early detection and in-time treatment. The studies conducted from 2012 to 2020 estimate that, by 2040, diabetes will affect about 642 million adults overall the world. To that end, DR will affect one from every three people with diabetes [2 ,3].

There are several lesions or signs of the DR, such as hemorrhages (HM), microaneurysms (MA), exudates (EX), venous reduplication (VR), neovascularization (NV), and venous loops (VL). The appearance of at least one of these lesions in the retina represents one of the DR grade [4]. In early grades, no symptoms are noted on the patient. On the other hand, in the progressive grades, patients may suffer from blurred vision, black areas in visions, floaters, distortion, progressive visual severity loss, and sudden blindness. DR can be categorized into non-proliferative DR (NPDR) and proliferative DR (PDR). NPDR includes mild, moderate, and severe grades. Severe NPDR leads to the PDR category.

**Fig. 1:**
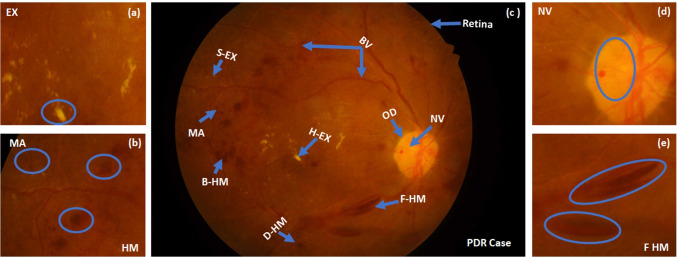
PDR contains most of DR lesions with different sizes, areas, features, and count on different regions on retina: (**a**) H-EX and S-EX, (**b**) B-HM and D-HM, (**c**) retina anatomy and PDR case, (**d**) NV from OD, and (**e**) F-HM

MA is the earliest clinical DR sign. It appears as small red dots on the BV. It may be increased in size in larger BVs. It appears in the retina’s superficial layers. Besides, it is accumulated by fibrin and red blood cell in its lumen. Another variation is the laceration. It produces blot/flame hemorrhages (B-HM and F-HM). Dot (D-HM) and B-HM occur as MA membranes in the retina’s deeper layers, such as the inner nuclear and outer plexiform. F-HM is superficial HM while B-HM is deeper [5]. HM appear similar to MA if they are small and vice versa. Moreover, EX is a vital DR sign and maybe soft (S-EX) or hard (H-EX). The occurrence of these lesions forms the different DR grades. The mild grade is diagnosed by appearing a little small MA, but HM’s occurrence with MA and soft EX refers to moderate grade. Increasing the number and regions of the aforementioned signs and/or H-EX leads to severe grade. Moreover, the closer the EX to the macula determines the grade of maculopathy and macular edema (ME). PDR grade means the growth of a new weak BV, which is called NV. In this grade, fragile and weak new BV forms on the retina’s surface. They result in blood leakage, which leads to blindness. Figure 1 shows most DR lesions’ appearance in the PDR case. Fundus scans are used widely in DR screening. It reports the retina abnormalities continuously. This modality includes various types, stereoscopic, and wide-field [6, 7]. We prefer to utilize it because it is inexpensive and can monitor DR progression over time [8].

Another critical thing to remember is that deep learning (DL), which has a vital critical role in diagnosing DR grades especially, convolution neural networks (CNN) that achieve great prosperity in many real-life applications, such as [4, 9, 10]. In general, human learners have inherent methods to transfer knowledge between tasks, especially if these tasks are correlated. The relevant knowledge from the previous learning experiences is recognized and applied while encountering new tasks. The more related new task is to the previous experience, the more easily it can be mastered [11]. Therefore, transfer learning uses knowledge from a learned task to improve the performance of a related task [12]. Transfer learning is needed when there is a limitation of the target training data. This could be due to the data being rare, expensive to collect and label, and inaccessible [13].

Despite the importance of detecting the DR disease early, many challenges threaten the ophthalmologists, radiologists, and developers, such as DR diagnosis need well-trained physicians. The manual detection of the retina’s abnormalities is time-consuming, inaccurate, and burdened by the physician [14]. On the other hand, the developed automated systems, which solve the manual detection problems, are based on hand-crafted features tools that burden the developers. These tools are sensitive to noise, contrast, and the illumination of the color fundus images, in addition to the variety and diversity of the extracted features. The few differences between the features that need to be extracted make it no easy task. A deep fine-tuned CNNs outperform the fully trained CNNs essentially, in a small training dataset [15]. Few studies diagnosed the DR grades. As reviewed in [5], 73% of the covered conducted studies detected only the presence/absence of DR, but just 27% of the studies are worked on various DR grades diagnosis [5].

With these problems in mind, we introduce a novel computer-aided diagnosis system (CAD) system based on transfer learning to accurately diagnose healthy and DR grades by utilizing color fundus images. The proposed system starts with some preprocessing operations. The system removes noise and enhances the contrast of the color fundus images. Normalization and some transformation processes were used to standardize the images sizes and maximize the limited datasets and avoid overfitting. In the modeling phase, we present a novel hybrid CNN model. The proposed model diagnoses the normal and various DR grades without the need to use hand-crafted feature extraction/selection and segmentation. We made a hybrid model that integrates the customized EyeNet model [16] and the fine-tuned DenseNet model [17] based on transfer learning. We modified the traditional EyeNet model to diagnose the normal and four various DR grades rather than DR presence/absence. Next, we optimized the hyperparameters and combined the customized EyeNet model with the DenseNet to fulfill the proposed E-DenseNet model. The proposed model incubated the accuracy of grading the DR cases from four various multi-label (ML) standard datasets. Each image in these datasets contains at least two DR lesions. We compared the proposed system with others and measured the performance by calculating significant performance metrics. The advantages of the two-hybrid models are reducing the complexity and ensuring robustness. Besides, it improves the generalization and the model inference ability. For reader convenience, the used abbreviations in this paper are listed in Table 1. The remainder of this paper is organized into five sections. Section 2 presents the related work. It discusses the current limitations and highlights the main directions and solutions that were included in the proposed system to overcome the current shortcomings. Section 3 explains the detailed phases and techniques, which were utilized in the proposed DL CAD system framework. Section 4 describes the different experiments and findings, which were conducted and got. Section 5 introduces the discussion and provides a comparative analytical study among the proposed CAD system and other state-of-the-art techniques. Finally, Section 6 presents a conclusion of our work and findings in addition to highlighting our future research directions.

**Table 1:** The used abbreviations

ACC	Accuracy	MA	Microaneurysms
AUC	Area Under Curve	ME	Macular Edema
AP	Average Pooling	MLC	Multi-Label Classification
APTOS 2019	Asia Pacific Tele-Ophthalmology Society	MLSVM	ML support vector machine
B-HM	Blot Hemorrhages	MP	max-pooling
BV	Blood Vessels	MSE	Mean Squared Error
BPs	bifurcation points	NPDR	Non-proliferative DR
CAD	Computer-Aided Diagnostic	NV	Neovascularization
CM	confusion matrix	OC	optic cup
CNN	Convolutional Neural Network	OCT	Optical Coherence Tomography
CONV	convolution	OCTA	OCT Angiography
CLAHE	contrast limited adaptive histogram equalization		
D-HM	Dot Hemorrhages	OD	Optic Disc
DL	Deep Learning	ON	Optic Nerve
DO	dropout	PA	Padding
DR	Diabetic Retinopathy	PDR	Proliferative DR
DSC	Dice Similarity Coefficient	PO	Pooling
EX	Exudates	QKS	Quadratic Kappa Score
FC	fully connected	ReLU	Rectified-Linear-Unit
F-HM	Flame Hemorrhages	RESNET	Residential Energy Services Network
FKM	fuzzy k-means	ROC	Receiver Operating Characteristic
FN	False Negative	ROIs	region of interest
FOV	Field of View	S	Stride
FP	False positive	S-EX	Soft Exudates
FRCNN	Fast Region-based CNN	SEN	Sensitivity
GANs	generative adversarial networks	SGD	Stochastic Gradient Descent
GAP	Global Average Pooling	SHAP	Shapley Additive exPlanations
GT	Ground Truth	SPE	Specificity
H-EX	Hard Exudates	TN	True Negative
HEBPDS	Histogram Equalization for Brightness Preservation Based on a Dynamic Stretching Technique	TP	True positive
HM	Hemorrhages	VGG	Very Deep Convolutional Networks
IDRiD	Indian diabetic retinopathy image dataset	VL	Venous Loops
$L_r$	Learning Rate	VR	Venous Reduplication

## Related work

Recently, many researchers have focused their attention on diagnosing the various DR grades depending on DL. They utilized DL techniques, such as pre-trained CNN models, to save the effort of extracting and selecting features as compared to the hand-crafted feature-based and segmentation techniques. For example, Khalifa et al. [22] utilized deep transfer CNN models to diagnose the grades of DR from APTOS 2019 dataset. They applied reflections around the x, y, and x & y axes, respectively, as data augmentation. They compared AlexNet, Residential Energy Services Network (Res-Net18), SqueezeNet, Very Deep Convolutional Networks (VGG16, VGG19), and GoogleNet. The authors proved that DenseNet achieved high accuracy (ACC). They utilized only one dataset and ignored noise removal. The authors made data augmentation to enlarge the dataset to avoid overfitting.

**Table 2:** A comparison of some current studies with respect to accuracy (ACC), specificity (SPE), sensitivity (SEN), Dice similarity coefficient (DSC), Quadratic Kappa Score (QKS) and the area under the curve (AUC)

Study	Year	Analysis	Methods	Dataset	Aug.	Performance
Maninis et al. [18]	2016	BV and OD segmentation	CNN	DRIVE, STARE for BV segmentation, DRIONS-DB, RIM-ONE (r3) for OD	No	DRIVE DSC 82.2%, STARE DSC=83.1%, DRIONS-DB DSC 97.1%, RIM-ONE (r3) DSC 95.9%
Islam et al. [19]	2018	MA detection, DR grading based MA	CNN	KAGGLE	Yes	QKS 85.1%, AUC 84.4%,SEN 98%, SPE 94%
Eftekhari et al. [20]	2019	MA segmentation	CNN	E-Ophtha-MA	Yes	SEN 80%
Gurani et al. [21]	2019	DR detection	CNN	KAGGLE	No	recognition rate 93%
Khalifa et al. [22]	2019	DR grading	DenseNet	APTOS 2019	Yes	ACC 97.7%
Hagos and Kant [23]	2019	DR detection	Inception-V3	KAGGLE	Yes	ACC 90.9%
Abdelmaksoud et al. [24]	2020	EX, MA, HM, BV segmentation and DR grading	matched filter with first order gaussian derivative, morphological operation and MLSVM	DRIVE, STARE, MESSIDOR, IDRiD	No	ACC 89.2%, AUC 85.20%, SEN 85.1%, SPE 85.2%,PPV 92.8%, DSC 88.7%
Patil et al. [25]	2020	DR grading	CNN	Kaggle, MESSIDOR	No	ACC 89.1%
Nazir et al. [26]	2020	OD, CD, HM, EX, and MA segmentation	FRCNN, and FKM	ORIGA, MESSIDOR, HRF, DiaretDB1	No	mAP 94%
Shah et al. [27]	2020	DR Detection	CNN	MESSIDOR	No	SEN 99.7%, SPE 98.5%, AUC 99.1%, QKS 0.95
Tymchenko et al. [28]	2020	DR Detection	ensemble of (EfficientNet, SE-ResNeXt50)	APTOS 2019	Yes	SEN 99%, SPE 99%, QKS 0.92
Abdelmaksoud et al. [29]	2021	EX, MA, HM, BV segmentation and DR grading	U-Net and MLSVM	DRIVE, STARE, MESSIDOR, IDRiD, ChaseDB1, HRF, DIARETDB1, E-ophtha, DIARETDB0	No	ACC 95.1%, AUC 91.9%, SEN 86.1%, SPE 86.8%, PPV 84.7%, DSC 86.2%
Aswathi et al.[30]	2021	DR grading	Inception-V3	MESSIDOR	Yes	ACC 78% (0,1), 69% (0,2), 61% (1,2), 62% (1,3), 49% (2,3), 32% (0,3)

To emphasize the importance of the preprocessing step, Patil et al. [25] recorded the ACC of the CNN model with preprocessing and without. They found that the ACC of the CNN model with preprocessing outperforms the other method. They introduced a customized CNN model by hyperparameters tuning to classify the DR grades. Their model included five convolution (CONV) layers. Each one was followed by a max-pooling (MP) layer. They added a flatten layer then two fully connected (FC) layers. Unfortunately, the authors achieved less ACC and fell in overfitting.

Nazir et al. [26] combined Fast Region-based CNN (FRCNN) and fuzzy k-means (FKM) techniques to segment EX, HM, MA, OD, and optic cup (OC). The last two signs are segmented for detecting Glaucoma and DR. The authors utilized FRCNN to detect and localize the disease using a bounding box. In contrast, they used FKM to extract the region of interest (ROIs) from the localized regions. Their work’s main advantage is that they segmented some indicators or signs of DR, maculopathy, and Glaucoma. However, they did not detect the BV abnormalities. BV segmentation is essential to detect DR, not only HM, EX, or MA. On the other hand, BV, EX, HM, and MA are not only the signs of DR, as illustrated in the previous section.

Shah et al. [27] utilized CNN architecture to detect referable DR. The authors started their framework by differentiating the retinal images from non-retinal ones. After that, they applied quality assessment and data augmentation techniques. Then, they detected the DR stage. Finally, they annotate the DR lesion on the color fundus image. The authors achieved good results in detecting severity and normal classes, but they did not accomplish a reasonable classification for mild and moderate grades. Their system could not differentiate between the mild and moderate cases, especially in the absence of H-EX and S-EX signs.

Eftekhari et al. [20] segmented MA signs from the color fundus images using the CNN model. They filtered the images by a median filter and made normalization, then subtracted the retinal image’s background. They utilized the CNN model to classify MA and non-MA pixels. They used two CNN models. The first one included three CONV layers, one MP layer followed each CONV layer, and three FC layers. The second model was deeper than the first one as it used five CONV layers, one MP layer followed each CONV layer, and three FC layers. Although they tried to solve the data imbalance issue by making data augmentation, they manually employed the utilized network architecture and its parameters by trial and error. The way they follow was time-consuming and error-prone.

Gurani et al. [21] used a multi-layer perceptron or feedforward of ANN through backpropagation to detect DR classes from color fundus images. Their network layers were CONV, MP, Rectified-Linear-Unit (ReLU), dropout (DO), FC, and classification using softmax. They used the quadratic kappa score (QKS) and sensitivity (SEN) for performance evaluation. They applied their method to the Kaggle dataset.

Islam et al. [19] developed a CNN model to detect the early stages of DR by allocating the MA lesion. The authors used a multi-layer CNN architecture followed by two FC and one output layer. They resized all images to get the same radius. Then, they subtracted the local average color. After that, the authors clipped the images to remove the boundary effect. They made some data augmentation operations, such as rotation, cropping, flipping, and transition. Their proposed network architecture composed of 18 layers with ($4\times 4$) kernel size of CONV and MP was ($3\times 3$). Two FC layers followed each CONV layer. The authors applied the ReLU activation function and L2 regularization. They used the objective function mean squared error (MSE) and the stochastic gradient descent (SGD) optimizer. The author applied the binary classification to address healthy and DR cases. On the other hand, they applied another binary classification to differentiate the low grade (mild) and the other high grades (moderate and severe). The authors directed their work based on binary classification and ignored the ML classification (MLC). It is advisable to utilize the ML idea to benefit from the correlation among labels to produce new labels. The label correlation can improve the classification results.

Maninis et al. [18] extracted OD and BV by CNN. They performed two feature map volumes. The first four finer stages and the same for the second coarser to segment OD, and BV, respectively. They utilized SGD with momentum. It is essential to extract the features of BV after removing the OD. But the authors did not detect the DR grades or DR presence/absence as BV is not enough to detect DR and its various grades.

Hagos and Kant [23] utilized Inception-V3 model to make a binary classification. They detected only two classes, healthy and unhealthy cases. The authors cropped the color fundus images and resized them to $300\times 300$. In modeling, they operated an SGD optimizer with $5\times 10^{-4}$ and cosine loss function. Although they made a binary classification, they achieved low ACC.

Abdelmaksoud et al. [24] presented a comprehensive CAD system for DR grades detection based on ML classification. They performed some preprocessing operations on different fundus image datasets. They then segmented the most famous four DR lesions by utilizing a matched filter with a first-order Gaussian derivative filter and morphological operations. They segmented EX, HM, BV, MA, and bifurcation points (BPs). After that, they extracted the GLCM and the lesions areas. The authors depended on the hand-crafted methods and classified the DR grades using the ML support vector machine (MLSVM) classifier. These hand-crafted feature extraction and classification burden the developer, especially when applied to high-dimensional datasets. Besides, they entered all the normal and DR images into the segmentation process. Their system produced five segmented images from each normal one. If the dataset includes 10 normal images, then at least their system produced $8\times 5$ useless segmenting images of BV, BP, EX, MA, and HM, where $8\times 3$ of EX, MA, and HM were black images. This resulted in some confusion to the ophthalmologists. On the other hand, it wasted space and memory with useless black images.

Tymchenko et al. [28] detected DR from color fundus images. They did some augmentation processes, such as zoom, horizontal and vertical flip, transpose, and rotation. Based on the pre-trained models, the authors made an ensemble of three CNNs. They utilized EfficientNet B4, EfficientNet B5, and SE-ResNeXt50. The authors used Shapley Additive exPlanations (SHAP) in order to ensure training the useful features. They used dropout and weight decay for regularization. The advantage of their method is that it increases generalization and reduces variance. They need to calculate SHAP for the whole ensemble, not only for a particular network. Moreover, they want more accurate hyperparameter optimization.

In addition, Abdelmaksoud et al. [29] combined DL with conventional methods for DR grades diagnosis. They optimized the CNN U-Net model for segmenting EX, BV, MA, and HM. They extracted more features and utilized more various datasets. They also used the MLSVM classifier for the final diagnosis. Although they achieved higher performance than [24], they needed to increase the performance by using DL to deal with most DR lesion features. On the other hand, the fundus images’ features have few differences between each other and are very near to be similar to the significant eye contents. So, it is essential to extract more DR lesions features, not only the most famous EX, BV, HM, and MA. The practical solution is to utilize an accurate DL technique to extract more feature maps from the entered fundus images without segmenting each lesion and extracting some features. Therefore, they developed our CAD system based on the proposed hybrid E-DenseNet model by utilizing transfer learning to diagnose the health and DR cases from various small and large Multi-Label (ML) datasets.

Aswathi et al. [30] utilized a pre-trained InceptionV3 on ImageNet to detect DR grades. They started their framework by enhancing the fundus images by using contrast limited adaptive histogram equalization (CLAHE) and Powerlaw transformation. They assigned DO to 0.5. They measured the performance of each class against the others. The authors compared InceptionV3, VGG19, ResNet, NASNet, and MobileNet. They found that all models approximately have equal ACC, but the VGG19 model takes a short running time. The main limitations of their work are that the model performance decreases with the increase of the number of classes or categories. Besides, their framework suggests better results in classifying normal and mild, but not much efficient in classifying (moderate, severe) and (normal, severe) binary classifications.

Table 2 lists the summary of the current literature that was conducted in DR diagnosing from the year 2016 to 2021. From the previous review, we can conclude the current literature’s main limitations in DR grading from color fundus images as follows:

- Most studies focused on detecting the presence or absence of DR. They ignored detecting the DR grades.
- Many studies utilized small and imbalanced datasets.
- Many studies could not predict the mild grade accurately, while the other could not differentiate between mild and moderate grades, especially with the absence of H-EX and S-EX.
- Some studies ignored preprocessing steps, while the noise and contrast affect the classification accuracy.
- Many systems fall with respect to the overfitting.

To overcome the current literature’s limitations and improve the diagnosis performance of detecting healthy and DR cases, we produce the CAD system based on the hybrid E-DenseNet DL model. Primarily, we preprocessed the images. The main goal is to enhance contrasts and remove the noise of the entered images. Then, we made some transformation processes to put all images in a standard size and increase their number. We resized images to ($256\times 256$), cropped, rotated, and made color normalization for all images of the four utilized ML datasets. Then, in the modeling phase, we customized the traditional EyeNet model [16] by optimizing its hyperparameters to diagnose the healthy and various DR grades accurately [16]. We combined the customized EyeNet model and the DenseNet BC-121 architecture [17] to produce the E-DenseNet model. We compared the proposed E-DenseNet model with some state-of-the-art models. In the comparison, we utilized five different performance metrics to guarantee the model performance in diagnosing the healthy and various DR grades.

## The proposed CAD system

**Fig. 2:**
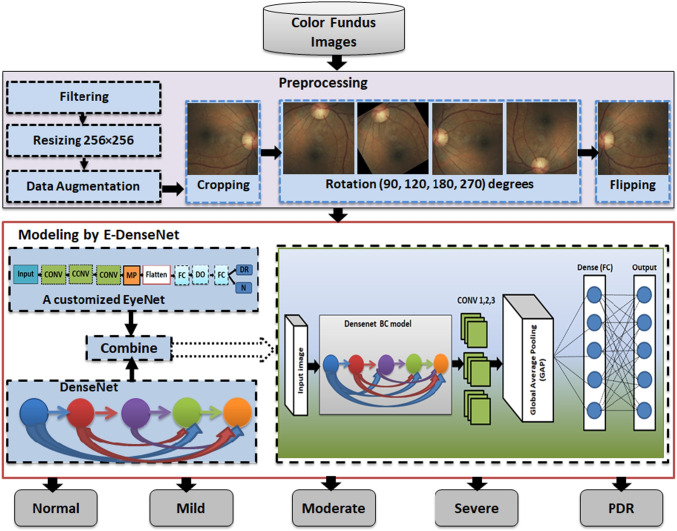
The proposed CAD system

This work is an extension to our work in [31]. In this section, we give a detailed explanation of the proposed framework. To diagnose the healthy and different DR grades, we built three phases framework. It starts by supplying the preprocessing phase with the four datasets, EyePACS [32], Indian diabetic retinopathy image dataset (IDRiD) [33], MESSIDOR [34], and Asia Pacific Tele-Ophthalmology Society (APTOS 2019) [35]. In the preprocessing phase, we care about enhancing the images and removing noise. After that, we scaled the images to a standard size and made some transformation processes, such as cropping, rotation, and mirroring. The normalized preprocessed images are fed to the customized E-DenseNet model in the modeling phase. Finally, we made the validation by training and testing the proposed model. Figure 2 shows the proposed CAD system. We present below the three phases of the proposed framework in detail.

### Preprocessing

We achieved the preprocessing phase by performing different steps and utilizing various techniques as follows:

- Filtering and contrast enhancement: This step is critical in most medical image analysis systems. The medical images are characterized by various noise, artifacts, and insufficient quality that vary from one modality to another. Fundus images suffer from illumination, low contrast and quality, and noise. In this respect, image quality has a large influence on model performance. If the contrast of the images is insufficient, the extracted features of the processed image will be insufficient. The enhancement of images can affirm the local or overall characteristics of the images, clear the unclear image, assure certain features of interest, suppress unnecessary features, and enlarge the difference between the features of the various objects in the images. Moreover, it can improve image quality, enhance image interpretation and recognition, fertilize information, make images more suitable for human visual systems, and reduce the training time [36]. We enhanced the contrast and filtered all images by histogram equalization for brightness preservation based on a dynamic stretching technique (HEBPDS) and median filter, respectively [24].
- Resizing: We resized all of the images to a standard size $256\times 256$.
- Data augmentation: It is crucial in our work to avoid overfitting in the utilized DL models. There are different transformation techniques, such as geometric, kernel filters, color space transformation, mixing images, random erasing, adversarial training, feature space augmentation, neural style transfer, generative adversarial networks (GANs) based augmentation, and meta-learning schemes [37]. In this phase, we made the data augmentation using data transformations, such as cropping, rotation, and flipping. By data augmentation processes, we increased the number of images by a factor of 5 times in addition to the resized and enhanced whole original images compared with the original dataset.
  1. Cropping: It means mixing the width and height dimensions by cropping the image’s central patch. It cut only the most significant part of the retina and remove the black contour and unneeded parts. We cropped the entered images to remove noise and unnecessary outliers in addition to focus on the retina part.
  2. Rotation: It means rotating the image right or left around an axis between 1^o^ and 359^o^. We rotated the cropped images to 90^o^, 120^o^, 180^o^, and 270^o^.
  3. Flipping: It means overthrowing the image horizontally or vertically.
  The imbalance of the datasets and the limited number of training images are solved by applying different data augmentation techniques. All black images that gave only black backgrounds without retina as in the EyePACS dataset were removed. Algorithm 1 shows the steps of the preprocessing phase.

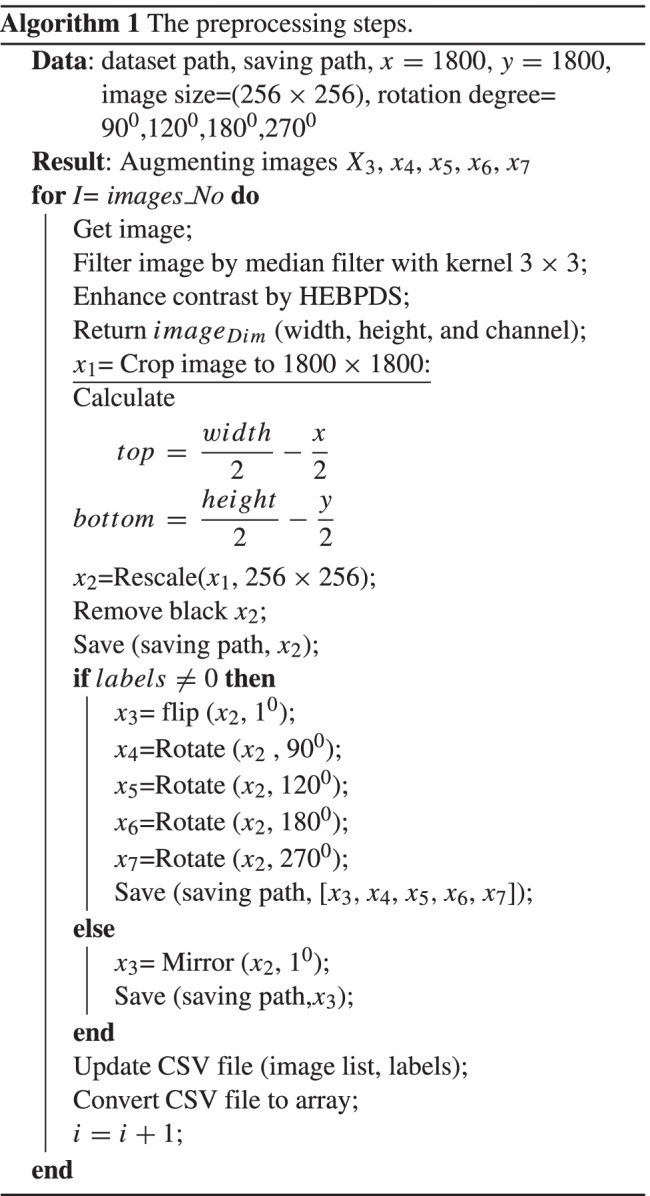

### Modeling

In this phase, we give an overall definition for transfer learning and highlight details about EyeNet [16] and the DenseNet-BC architectures [17]. In addition, we provide the pseudo-code of the modeling steps.

#### Transfer learning

CNN is a type of DL architecture for analyzing data, especially images. It gives better results in image ML classification. It consists of three basic layers: CONV, PO, and FC [38]. The first and second layers perform feature extraction and reduction while the third layer maps the final output’s extracted features. The main advantage of using these methods is that CNN does not require hand-crafted feature extraction. Many CNN models are commonly used. These architectures can be categorized into classical and modern neural networks (NNs). The classic NNs are like LeNet-5, AlexNet, and VGG16. The modern NNs are like Inception, ResNet, ResNetXt, and DenseNet [39].

Fine-tuned DL architectures are helpful in medical image analysis. They can outperform the fully trained CNNs, especially in small training set [11]. We give the full definition of transfer learning as the following. The pre-trained models on a particular task can be applied to other tasks. This is the idea of transfer learning. Assume that we define the domain by feature space and the probability distribution where *D*, *F*, and *P*(*F*) are for the domain, feature space, and the probability distribution, respectively.

1 $$\begin{aligned} F = [f_{1} , ... , f_{n}] \in F \end{aligned}$$

For the given domain *D*, the task *T* is defined by a label space *Y* while the predictive function is *pf*(.). It is learned from the feature vector and label pairs ${f_{i} , y_{i} }$ where $f_{i} \in F$ and $y_{i} \in Y$. Now, $D = [F , P(F)]$ and $T = [ Y, pf(.)]$, but the source domain $D_{S}= [(f_{S1}, y_{S1}) ... , (f_{Sn}, y_{Sn})]$, where $f_{Si} \in F_{S}$ is the $i_{th}$ data instance of $D_{S}$ and $y_{Si} \in Y_{S}$ is the corresponding class label for $f_{Si}$.

In the same way, we can use the same learned task on other domain or target domain. Let $D_{T}$ is defined as the target domain data and $DT = [(f_{T1}, y_{T1}) ... , (f_{Tn}, y_{Tn})]$, where $f_{Ti} \in F_{T}$ is the $i_{th}$ data instance of $D_{T}$, and $y_{Ti} \in Y_{T}$ is the corresponding class label for $f_{Ti}$. Furthermore, the source task is notated as $T_{S}$, the target task as $T_{T}$, the source predictive function as $f_{S}(.)$, and the target predictive function as $f_{T}(.)$. Now, by given the $D_{S}$ with related $T_{S}$ and $D_{T}$ with the related $T_{T}$. In conclusion, we can define the transfer learning as the improvement of the target predictive function $f_{T}(.)$ where $D_{S} \ne D_{T}$ or $T_{S}\ne T_{T}$.

#### The EyeNet model

**Fig. 3:**
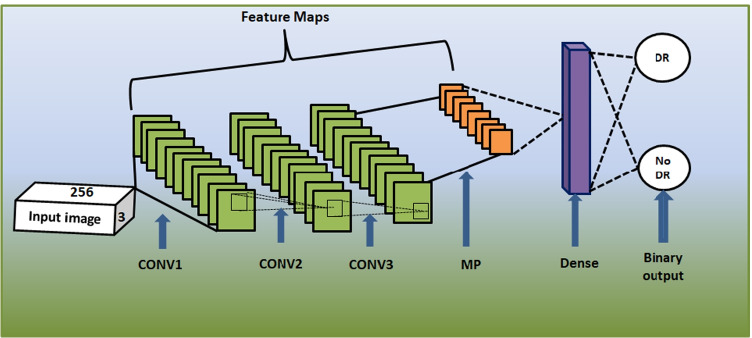
The traditional EyeNet model

The main advantage of utilizing the traditional EyeNet model is its ability to diagnose the severe or PDR grade in imbalanced datasets accurately. Another advantage of EyeNet is that its suitability in working on large-scale datasets. Besides, its ability to perform the diagnosis on local devices. Therefore, the model can be facilely used in remote areas. The model architecture includes 3 CONVs layers with 32 filters in each layer, followed by MP with $2 \times 2$ and ended by a single FC layer with size 128. The softmax classifier outputs two nodes (DR, normal). The utilized activation function, optimizer, loss function, kernel size, stride, DO, batch size are sigmoid, Adam, binary cross-entropy, $8 \times 8$, 1, 0.2, and 512 with 30 epochs, respectively. Figure 3 shows the traditional EyeNet architecture.

#### The customized EyeNet model

We modified the traditional EyeNet to output five classes instead of only two classes (normal, DR) from ML datasets. On the other hand, we enhanced the performance of the EyeNet as the model became deeper. Besides, we optimized the hyperparameters, such as L regularization, learning rate ($L_r$), DO, and optimizer. We utilized ReLU activation for non-linearity and extracting complex features. Moreover, ReLU is more efficient in computations than sigmoid and does not vanish gradient. Three CONV layers with a kernel size of $4 \times 4$ are added. The used parameters are $L_r$ of $9\times 10^{-5}$, L1 is $le-6$, L2 is $le-5$, pool size is $4 \times 4$ with stride equals 1, DO is 0.5, the optimizer is Adam with $L_r=5\times 10^{-5}$, loss function is categorical cross-entropy and softmax classifier with 200 epochs. The PO layer manages the feature maps dimensions and controls the overfitting. DO, $L_1$, and $L_2$ also controls the overfitting. Algorithm 2 shows the steps of the customized EyeNet model.

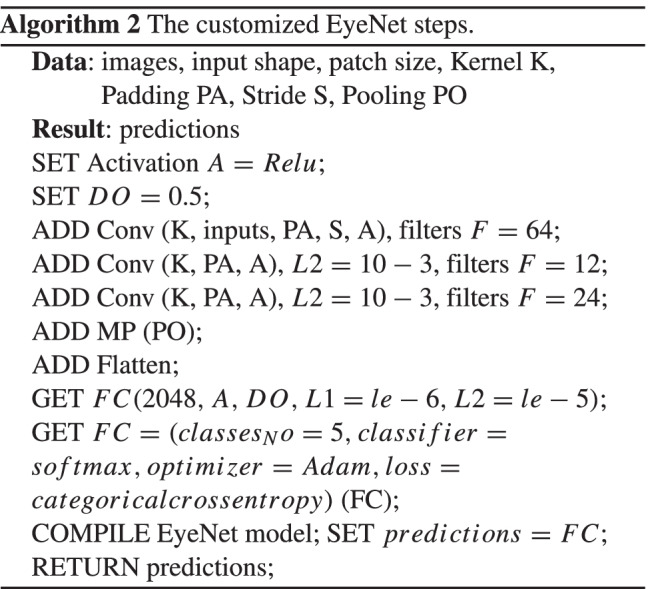



#### The DenseNet model

It works like ResNet [39, 40] but it concatenates the output of one layer with the incoming feature maps of the previous one rather than summing them. It connects the layer output with the following one after some transformation operations, such as CONV, PO, BN, and ReLU activation. The main features of the DenseNet models are the following: (1) The network is narrow and easier, (2) It uses few filters and requires fewer parameters, in addition to the efficiency of used parameters, (3) It lessens the redundant feature maps and saves the memory space, (4) DenseNet helps the final classifier to make its decision on all feature maps in the network [17], and (5) All layers can easily access their preceding layers. Therefore, it helps in reusing the information from the previously calculated feature maps easily.

**Fig. 4:**
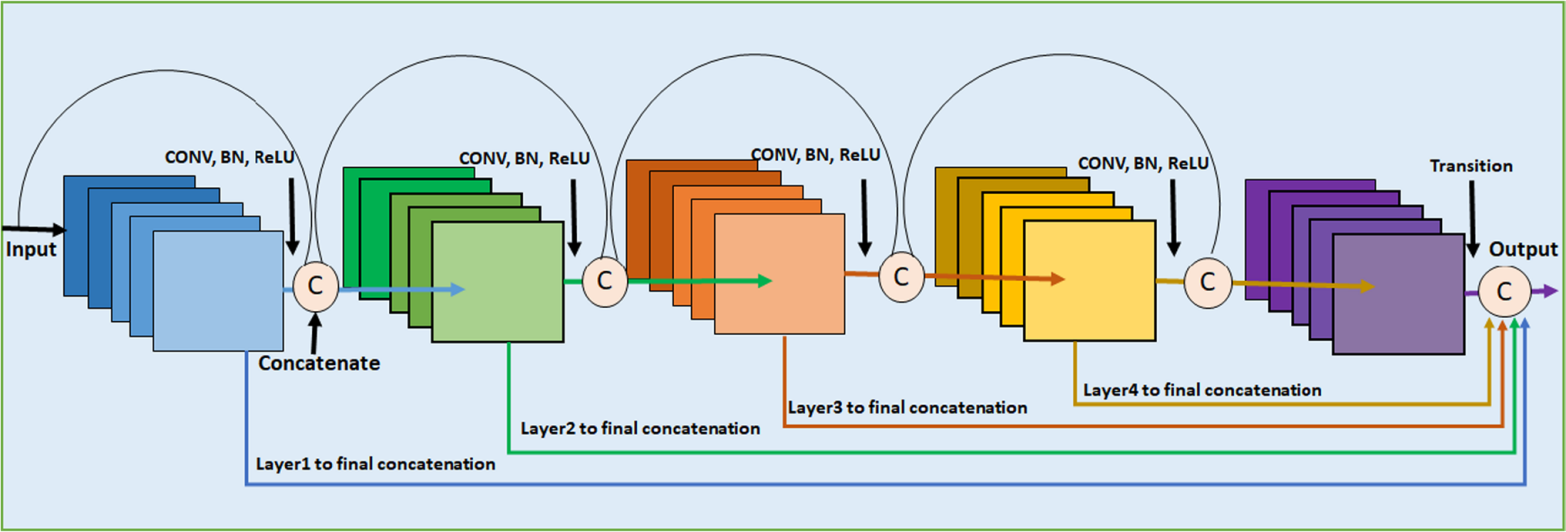
The DenseNet model

The different types of DenseNets are DenseNets-B, DenseNets-C, and DenseNets-BC. DenseNets-B are just regular DenseNets. They decrease the feature map size by getting merits of CONV layer with filter $1\times 1$ before CONV layer with filter $3\times 3$. They improve the efficiency of the computations. DenseNets-C are considered little incremental step to DenseNets-B [41]. Figure 4 presents the DenseNet architecture.

Let $x_{L}$ is the output of the $L_{th}$ layers, $T_{L}(xL)$ the nonlinear transformation processes, such as batch normalization, ReLU activation, and CONV layer with filter $3\times 3$ which are done to the output of the previous layer $x_{L-1}$. In concatenating all the features maps through the feedforward way, the output of the $x_{L}$ is defined by Eq. 2.

2 $$\begin{aligned} x_{L}= T_{L}([x_{L-1}, x_{L-2}, x_{L-3}, \ldots .,x_{0}]) \end{aligned}$$

The layer between each dense block is called the transition layer that performs CONV and average pooling (AP). Each $T_{L}(X_{L})$ for each layer produces feature maps *K*, and the input feature map is $K_{0}$, which is determined by the number of channels of the input image. On the other hand, *K* is called the growth rate of the network. So, it should be minimized to a small integer. Therefore, the layer $L^{\mathrm {th}} = K\times (L-1) +k_{0}$. In order to reduce the input feature maps, the bottleneck layer is added. It is represented by adding CONV layer with filter $1\times 1$ in the $T_{L}$ processes before each CONV layer with filter $3\times 3$, such as (BN, ReLU, and CONV layer with filter $1\times 1$) then (BN, ReLU, and CONV layer with filter $3\times 3$). This layer improves computation efficiency. The model, in this case, is called DenseNet-B. This model can reduce four times *K*. On the other side, to reduce the generated features maps from the transition layer to improve the compactness, the compression factor $\theta$ is used as follows:

- If the dense block contains features maps *m*, then the transition layer produces $\theta m$ where $0<\theta \le 1$.
- If $\theta =1$, then the transition layer produces m without any changing.
- If $\theta <1$ then the model is called DenseNet-C. The model becomes DenseNet-BC [17, 39].
- If both the bottleneck and transition layers with $\theta < 1$ are used. We conclude that the DenseNet-BC model reduces the generated features maps from the bottleneck and transition layers. Therefore, it is more efficient than DenseNet-B and DenseNet-C

#### The proposed E-DenseNet model

**Fig. 5:**
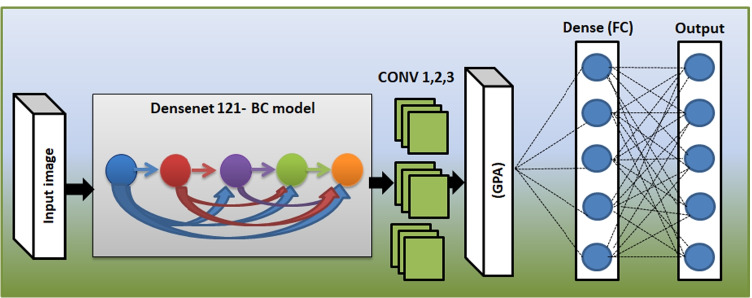
The proposed E-DenseNet model

Figure 5 shows the proposed E-DenseNet architecture. We applied dense block based on the fine-tuning DenseNet-BC 121, while 121 denotes the model depth. Then, we added three CONV layers from the customized EyeNet. After that, we insert global average pooling (GAP), which does not need any parameters to process and prevent overfitting. Finally, FC outputs five healthy and DR grades. The parameters that were optimized here were, such as optimizer Adam with $L_r= 10^{-4}$ and $decay=1e-6$, loss function was categorical cross-entropy, activation function was ReLU, and softmax classifier. We utilized regularization $L_2= 10^{-4}$ with 100 epochs, and $DO=0.5$. By utilizing the $L_2$ regularization, DO, and data augmentation, we can avoid overfitting. Algorithm 3 shows the pseudo-code of the proposed E-denseNet model.

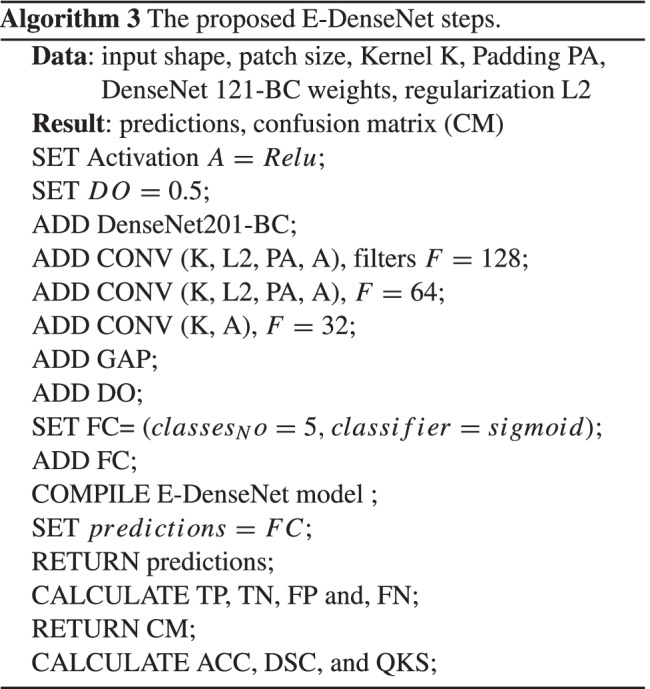



## Experimental results

This section gives a detailed demonstration of the four utilized ML datasets, which are APTOS 2019, MESSIDOR, EyePACS, and IDRID. After that, we present the detailed experiments and the final proposed CAD system results based on the proposed E-DenseNet model due to five performance measures. On the other side, we compared the proposed system with other models. The utilized datasets description is shown in Table 3. Table 4 shows the class distributions on the four used datasets from class 0: normal to class 4: PDR.

### Dataset description

**Table 3:** The main specifications of the four utilized benchmark datasets

Dataset	Images	Camera	Resolution	Format	GT	Experts	Classes
APTOS 2019 [35]	18590	different cameras with various FOV	from $474 \times 358$ to $3388 \times 2588$	JPG	Yes: grading CSV	many	5
IDRiD [33]	597	AKowa VX-10 alpha with $50^{\mathrm {o}}$ FOV	$4288 \times 2848$	JPG	Yes: grading CSV	unknown	5
EyePACS [32]	35,126	different cameras with various FOV	$1440\times 960$	jpeg	Yes: grading CSV	many	5
MESSIDOR [34]	1200	Topcon TRC NW6 with $45^{0}$ FOV	$1440\times 960$	TIFF	Yes: grading CSV	3	4

**Table 4:** Classes of the utilized datasets: 0 = Normal, 1 = mild, 2 = moderate, 3 = severe NPDR, and 4 = PDR

Dataset	0	1	2	3	4	Train	Valid
EyePACS	25810	2443	5292	873	708	34126	1000
MESSIDOR	32	13	11	−	44	80	20
IDRiD	134	20	136	74	49	330	83
APTOS 2019	1805	370	999	193	295	2929	733

- EyePACS dataset [32]: It is a large set of retina images with high-resolution. These images were captured under various imaging conditions, such as various camera types and settings with different sizes and appearances. Images are captured from different people for paired (right and left) eyes. Clinicians have rated DR in each image on a scale of 0 or normal to 4 or PDR cases. However, there are many black images contained in this dataset. All images were stored in JPEG format.
- APTOS 2019 dataset [35]: It includes about 18590 color fundus images. They are separated into 3662 images for training, 1928 images for validation, and 13000 images for testing. The JPG is the extension of all images. The ground truth (GT) of the dataset is two CSV grading files for training and testing.
- MESSIDOR dataset [34]: The color fundus images were captured in three different sizes, which are $1440 \times 960$ with 8 bits color plane. It includes 1200 images.
- IDRiD dataset [33]: It contains 516 fundus images, which were captured for the DR grading. They are in JPEG format. The images are split into 413 images for training and 103 images for testing sets. They have a large resolution of $4288\times 2848$. All images were captured by the same digital fundus camera, which is AKowa VX-10 alpha with $50^{0}$ FOV. The camera was centered near the macula.

### The Performance Measures

We utilized five different performance measures, which are ACC, SEN, SPE, DSC, and QKS. ACC is the ratio of the correct predictions to the total number of the input samples. Of course, ACC works well if the numbers of samples belonging to each class are equal. Therefore, we utilized DSC, which is essential in imbalanced dataset evaluation. To define the equations of the aforementioned performance measures, we first define their arguments, which are true positive (TP), true negative (TN), false positive (FP), and false negative (FN). TP is the cases that are predicted true, and their actual output is also true. TN is the cases that are predicted false, and their actual output is also false. FP is the cases that are predicted true, and their actual output is false. Finally, FN is the cases that are predicted false, and their actual output is true. Each argument is determined for each class against the rest of the classes. We mean that TP, TN, FP, and FN are evaluated for each category of classes separately. For example, we constructed CM including true labels versus predicted labels. The values are reported for class 0/class 1, class 0/class 2, class 0/class 3, and class 0/class 4, and so on for classes 1, 2, 3, and 4, as will be illustrated next.

DSC determines how many samples are classified correctly. SEN asks about how many DR cases are correctly predicted.

On the contrary, SPE asks about how many normal cases are correctly predicted [42]. Finally, QKS is a measure of the agreement between two raters (the human scores and the prediction scores). These raters determine which category some samples belong to. The two raters either agree in their rating or disagree by subtracting the agreement according to chance. QKS falls between -1 (which means a complete disagreement between the raters) and 1 (which means a complete agreement between the raters). ACC, SEN, SPE, DSC, and QKS performance measures matrices can be calculated from Eqs. 3–7.

3 $$\begin{aligned} ACC= & {} \frac{TP+TN}{TP+TN+FP+FN}\end{aligned}$$

4 $$\begin{aligned} SEN= & {} \frac{TP}{TP+FN} \end{aligned}$$

5 $$\begin{aligned} SPE= & {} \frac{TN}{TN+FP}\end{aligned}$$

6 $$\begin{aligned} DSC= & {} \frac{2\times TP}{2\times TP +FP+FN}\end{aligned}$$

7 $$\begin{aligned} QKS= & {} \frac{p_{a} - p_{e}}{1 - p_{e}} \end{aligned}$$

To calculate QKS, suppose $p_{a}$ is the ratio of observations in agreement, $p_{e}$ is the ratio in agreement due to chance. The relationship between SEN, SPE, and QKS is defined with details in [43]. For the relation between SEN and QKS, SEN is linearly increased as QKS increases. On the other hand, SPE linearly increases also as QKS increases.

### The results

We implemented the proposed framework by using python 3.7 and cloud computing “Google Colab”. This work was implemented on TensorFlow 2.4. Also, for the preprocessing steps, we utilized the open-source Python library OpenCV. For classification, we utilized DL Python open-source Library (Tf) Learn. We ran our experiments on a core i5/2.4 GHz machine. It had 8GB RAM and an NVIDIA VGA card with 1GB VRAM.

Table 5 shows the hyperparameters optimization experiments of the EyeNet model on the EyePACS dataset. We combined the customized EyeNet with the DenseNet-BC 121 to accurately diagnose the normal and the DR grades from different color fundus images. The regularization L1 and L2 in addition to DO and AP avoid the overfitting.

**Table 5:** The customized EyeNet hyperparameters to diagnose the DR grades on EyePACS dataset

Optimizer	Parameters	Epochs	ACC (%)	DSC (%)	QKS
Adam	$L_r=0.01$	20	67.56	76.60	0.578
Adam	$L_r=0.01$	50	66.40	78.33	0.615
Adam	$L_r=0.01$	75	68.03	75	0.55
Adam	$L_r=0.01$	100	67.75	72	0.52
SGD	$L_r=le-3$	100	64.35	66.60	0.458
RmsProp	$L_r=le-4$	100	70.12	71.6	0.532
Adagrad	$L_r=le-4$	100	62.50	75	0.56
Adam	$L_r=0.01$	120	66.20	68.33	0.47
Adam	$L_r=0.001$	150	71.00	76.60	0.594
Adam	$L_r=0.001$	175	71.40	80	0.646
Adam	$L_r=0.0001$	50	74.50	81.60	0.681
Adam	$L_r=2\times 10^{-4}$	50	70.70	78.30	0.621
Adam	$L_r=0.001$	100	74	83	0.704
Adam	$L_r=0.001$	150	70	75	0.58
Adam	$L_r=5\times 10^{-5}$	200	76	83.30	0.713
Adam	$L_r=7\times 10^{-5}$	200	78.80	83.30	0.719
Adam	$L_r=9\times 10^{-5}$	200	79.50	85	0.745
Adam	$\beta _1=0.2$, $\beta _2=0.2$, $\epsilon =1e-08$, $decay=0.01$,$L_r=10^{-4}$	200	70	73	0.55
Adam	$decay=le-6$, $L_r=10^{-5}$	200	95.5	95	90.1
Adamax	$\beta =(0.9, 0.999), \epsilon =1e-08$, $L_r=0.002$	10	65.2	62	30
Adadelta	$\rho =0.9, \epsilon =1e-06, L_r=0.001$	100	74	68	20
Adagrad	$\epsilon =1e-10, L_r=0.001$	50	66	73	15.7

From Table 5, we can notice that the customized EyeNet model with the aforementioned hyperparameters achieved 95.5%, 95%, and 90.1 for ACC, DSC, and QKS, respectively. We applied the customized EyeNet model on the other three datasets.

In addition, we present the results of applying the proposed CAD system based on the E-DenseNet model and others, such as the customized EyeNet, ResNet50 [40], Inception V3 [44], VGG19 [45] on the four benchmark ML datasets. On the other hand, we experimented E-DenseNet-BC with depths of 169, 121, and 201 by the customized pre-trained weights and the pre-trained ImageNet weights.

In APTOS 2019 dataset, the customized EyeNet model achieved 75.7%, 74.9%, and 0.609 for ACC, DSC, and QKS, respectively.

**Table 6:** The comparisons between the customized EyeNet, ResNet50 [40], Inception V3 [44], VGG19 [45], and the proposed E-DenseNet BC with different depths and weights on APTOS 2019 dataset due to ACC, SEN, SPE, DSC, QKS, calculation time (T) in minutes (m) performance measures

Model	ACC (%)	SEN (%)	SPE (%)	DSC (%)	QKS	T(m)
A customized EyeNet	75.7	76	82	74.9	0.61	14
ResNet50 [40]	67.4	70	52.6	66.2	0.51	27
Inception V3 [44]	49.3	53	51	49.1	0.13	38
VGG19 [45]	72.5	80	68	71	0.55	17
E-DenseNet BC-169-ImageNet	80.2	73.6	92	80	0.70	5.2
E-DenseNet BC-169	80.6	72	90	80.9	0.71	7
E-DenseNet BC-201-ImageNet	82.2	74.6	92.3	82	0.73	10
E-DenseNet BC-121-ImageNet	72	75	48.4	71.54	0.58	3
E-DenseNet BC-121	84	94	73	83.7	0.75	4

**Fig. 6:**
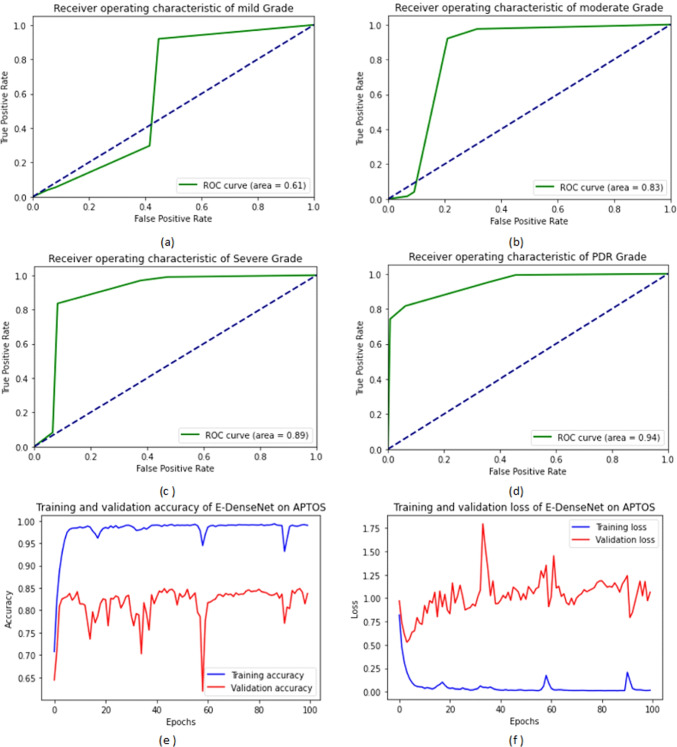
The ROC curves areas of the DR grades and the training and validation ACC and loss on APTOS 2019 dataset: (**a**) the ROC curve of the mild grade, (**b**) the ROC curve of the moderate grade, (**c**) the ROC curve of the severe NPDR grade, (**d**) the ROC curve of the PDR grade, (**e**) the training and validation ACC, and (**f**) the training and validation loss

From Table 6, we observe that the proposed E-denseNet BC-121 with the pre-trained customized weights model achieved higher ACC, DSC, and QKS. It ranked the first order, while the same model with 201 depth using the ImageNet weights came in the second order. The proposed model with 169 depth with both customized and ImageNet weights came in the third order. The customized EyeNet came in the fourth order with a difference of 6%, 18%, 9%, 6.9%, and 0.11 for ACC, SEN, SPE, DSC, and QKS, respectively. VGG19 came in the fifth order and ResNet50 in the sixth order. Finally, Inception V3 came in the last order. There was no agreement between the raters. E-DenseNet BC-169 with the pre-trained ImageNet and customized weights were very near each other. They were still greater than E-DenseNet BC-121 architecture with the customized pre-trained weights in SPE while lower in SEN than E-DenseNet BC-121. For the calculation time comparison, we can observe that the InceptionV3 model takes a long time followed by ResNet50, then VGG19 with a difference of three minutes (m) more than the customized EyeNet model. The calculation time is 38m, 27m, 17m, and 14m, respectively. On the other hand, the E-DenseNet models take 10m, 7m, 5.2m, 4m, and 3m for E-DenseNet BC-201-ImageNet, E-DenseNet BC-169, E-DenseNet BC-169-ImageNet, E-DenseNet BC-121, and E-DenseNet BC-121-ImageNet, respectively. Figure 6 shows the receiver operating characteristic (ROC) curves of the four DR grades in addition to the training and validation ACC and loss on APTOS 2019 dataset. We can notice that the system achieved a higher ROC curve area in PDR grade, severe NPDR grade followed by moderate grade, and a less ROC curve area in mild grade. The proposed system achieved 94%, 89%, 83%, and 61%, respectively.

**Table 7:** The comparisons between the customized EyeNet, ResNet50 [40], Inception V3 [44], VGG19 [45], the proposed E-DenseNet BC with different depths and weights on EyePACS dataset due to ACC, SEN, SPE, DSC, QKS, calculation time (T) in minutes (m) performance measures

Model	ACC (%)	SEN (%)	SPE (%)	DSC (%)	QKS	T(m)
A customized EyeNet	95.5	95.7	73	95	0.90	22
ResNet50 [40]	79.2	83	53	86.7	0.78	43
Inception V3 [44]	72.6	76.7	61	82	0.65	55
VGG19 [45]	82.3	87	49	80.6	0.69	45
E-DenseNet BC-169	86	90	55	93.3	0.89	8
E-DenseNet BC-169-ImageNet	83	87	61	91.6	0.86	7
E-DenseNet BC-201-ImageNet	90.6	94.3	53	95	0.92	9
E-DenseNet BC-121-ImageNet	88.1	93	68	93	0.89	5
E-DenseNet BC-121	96.8	98.3	72	98.3	0.97	5

From Table 7, we predestine that the proposed E-DenseNet BC-121 with the pre-trained customized weights achieved 96.8% for ACC, 98.3% for SEN, 98.33% for DSC and 0.97 for QKS. However, it achieves less SPE. The customized EyeNet came in the second order. It achieved 95.5%, 95.7%, 95%, and 0.90 for ACC, SEN, DSC, and QKS, respectively. The E-DenseNet BC-201-ImageNet ranked the third order, then, E-DenseNet BC-121-ImageNet followed by E-DenseNet BC-169 and E-DenseNet BC-169-ImageNet model. VGG19 model achieved higher ACC than ResNet50 by 3.1% difference, but ResNet50 was higher than VGG19 in DSC and QKS. It achieved 86.7% and 0.78, respectively. Inception-V3 model achieved less ACC, DSC, and QKS than ResNet50 by about 6.6%, 4.7%, 0.13, respectively. Although Inception V3 achieved higher DSC than VGG19 by 1.4%, VGG19 increased ACC and QKS by 10.3% and 0.04 from that war achieved by Inception V3, respectively.

For the calculation time comparison, we can observe that the InceptionV3 model still takes a long time, followed by VGG19, ResNet50, then, customized EyeNet model. The calculation time is 55m, 45m, 43m, and 22m, respectively. On the other hand, the E-DenseNet models take 9m, 8m, 7m, and 5m for E-DenseNet BC-201-ImageNet, E-DenseNet BC-169, E-DenseNet BC-169-ImageNet, E-DenseNet BC-121, respectively. E-DenseNet BC-121 is equal to E-DenseNet BC-121-ImageNet in calculation time.

**Fig. 7:**
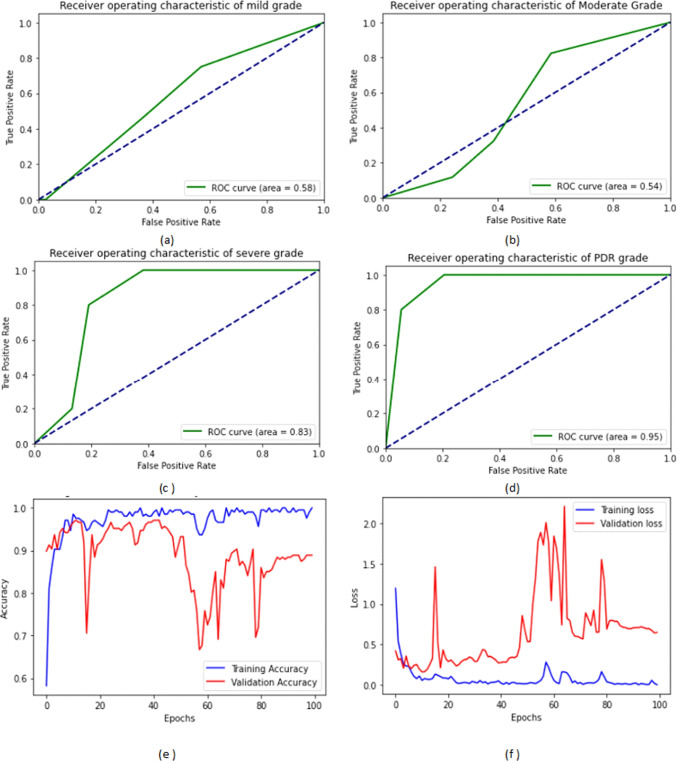
The four ROC curves of the four DR grades and the training and validation ACC and loss on IDRiD dataset: (**a**) the ROC curve of the mild grade, (**b**) the ROC curve of the moderate grade, (**c**) the ROC curve of the severe NPDR grade, (**d**) the ROC curve of the PDR grade, (**e**) the training and validation ACC, and (**f**) the training and validation loss

Figure 7 shows the ROC curves of the four DR grades in addition to the training and validation ACC and loss on the IDRiD dataset. We noticed that the AUC under the ROC curve for PDR, severe, mild, and moderate NPDR grades were 95%, 83%, 58%, and 54%, respectively.

**Table 8:** The comparisons of the customized EyeNet, ResNet50 [40], Inception V3 [44], VGG-19 [45], and the proposed E-DenseNet BC with different depths and weights on MESSIDOR dataset due to ACC, SEN, SPE, DSC, QKS, and calculation time (T) in minutes (m) performance measures

Model	ACC (%)	SEN (%)	SPE (%)	DSC (%)	QKS	T (m)
A customized EyeNet	63	62.5	90.8	63	0.48	15
ResNet50 [40]	37.5	38	22	38	0	33
Inception V3 [44]	37.5	38	40	38	0	50
VGG19 [45]	43.7	44	37	44	0	28
E-DenseNet BC-169-ImageNet	38	38	21	37	0.09	4
E-DenseNet BC-169	62.5	63	76	61	0.44	4
E-DenseNet BC-121-ImageNet	69.2	70	90	68.7	0.53	2
E-DenseNet BC-201-ImageNet	50.2	52	80	51.5	0.11	4
E-DenseNet BC-121	91.6	95	58	95.1	0.92	2

**Fig. 8:**
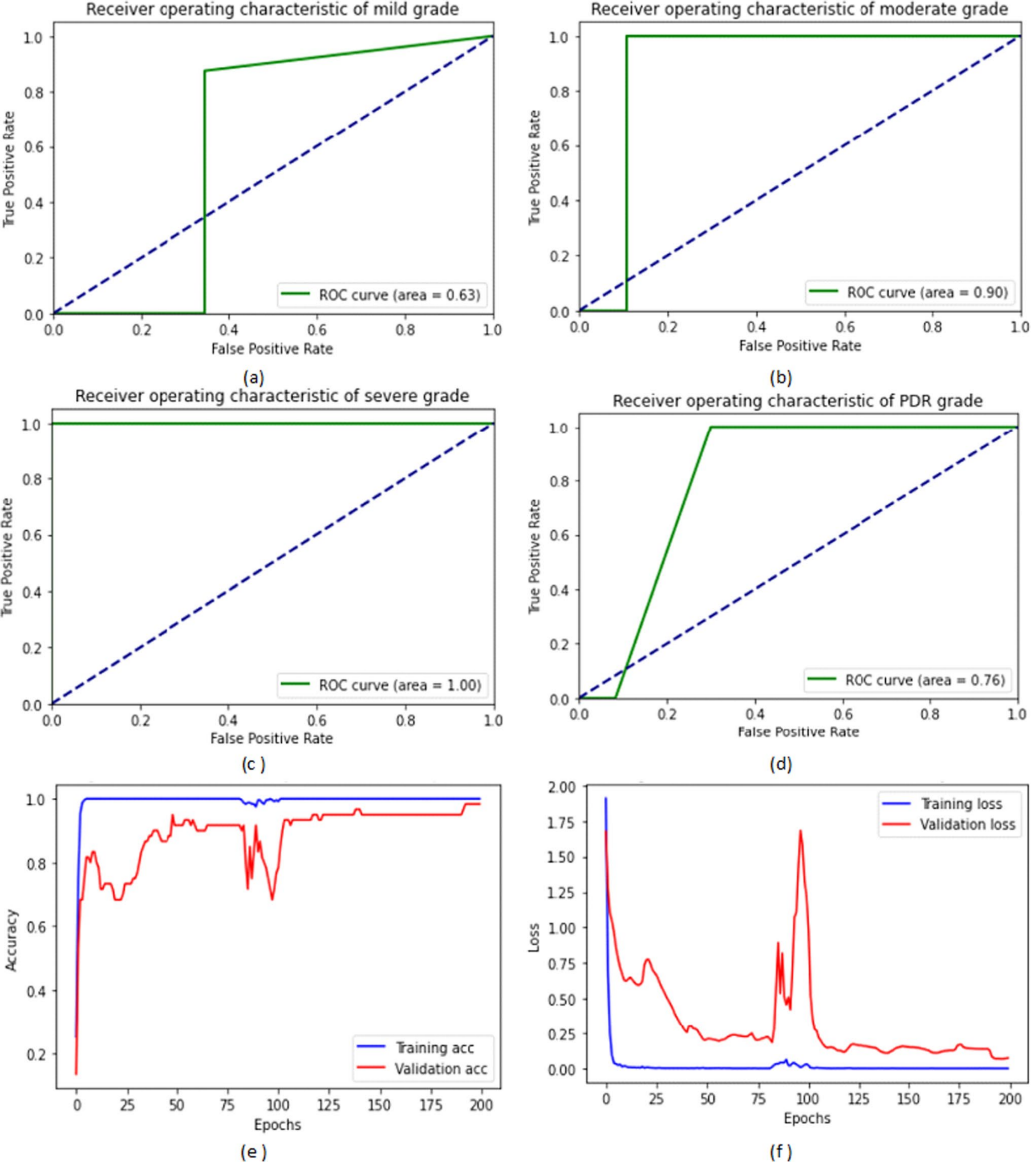
The four ROC curves of the four DR grades and the training and validation ACC and loss on EyePACS dataset: (**a**) the ROC curve of the mild grade, (**b**) the ROC curve of the moderate grade, (**c**) the ROC curve of the severe NPDR grade, (**d**) the ROC curve of the PDR grade, (**e**) the training and validation ACC, and (**f**) the training and validation loss

Figure 8 shows the ROC curves of the four DR grades in addition to the training and validation ACC and loss on the EyePACS dataset. We can notice that the system achieved a higher ROC curve area in severe grade, moderate NPDR grade followed by PDR, and achieved less ROC curve area in mild grade. The proposed system achieved 100%, 90%, 76%, and 63%, respectively.

**Fig. 9:**
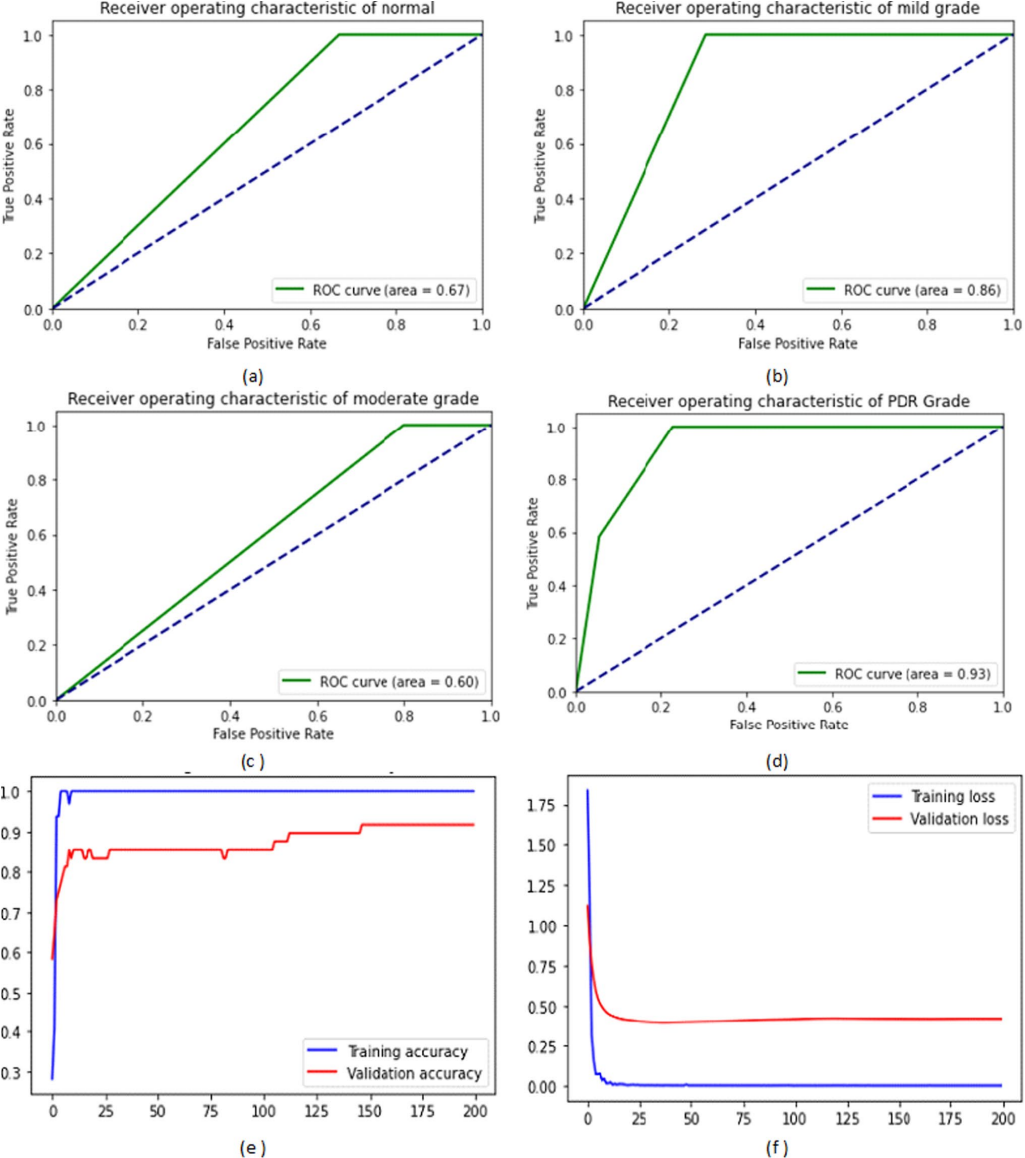
The four ROC curves of the four DR grades and the training and validation ACC and loss on MESSIDOR dataset: (**a**) the ROC curve of the normal cases, (**b**) the ROC curve of the mild grade, (**c**) the ROC curve of the moderate grade, (**d**) the ROC curve of the severe grade, (**e**) the training and validation ACC, and (**f**) the training and validation loss

**Table 9:** The comparisons between the customized EyeNet, ResNet50 [40], Inception V3 [44], VGG-19 [45], and the proposed E-DenseNet BC with different depths and weights on IDRiD dataset due to ACC, SEN, SPE, DSC, QKS, and and calculation time (T) in minutes (m) performance measures

Model	ACC (%)	SEN (%)	SPE (%)	DSC (%)	QKS	T(m)
A customized EyeNet	45	63	35	44.5	0.24	17.05
ResNet50 [40]	32.5	38	0	32.5	0	23.5
Inception V3 [44]	32.5	40	22	32.5	0	27.5
VGG19 [45]	33	40	22	32.5	0	16.4
E-DenseNet BC-169-ImageNet	66.3	70	49	66	0.53	6
E-DenseNet BC-169	61.4	70	43	60	0.46	4
E-DenseNet BC-121-ImageNet	64.2	61.3	50	63.8	0.49	3
E-DenseNet BC-201-ImageNet	62.2	61	55	61.1	0.48	7
E-DenseNet BC-121	93	96.7	72	96	0.94	3

Figure 9 shows the ROC curves of the normal and the three DR grades in addition to the training and validation ACC and loss on the MESSIDOR dataset. We can notice that the system achieved a higher ROC curve area in PDR, and mild NPDR grades. It achieved 93%, and 86% respectively.

Table 8 shows the comparison between the nine models on the MESSIDOR dataset. We can notice that the proposed E-DenseNet BC-121 architecture model achieved higher ACC, SEN, SPE, DSC and QKS than other models. It achieved 91.6%, 95%, 95.1%, and 0.92, respectively. E-DenseNet BC-121-ImageNet came in the second order, then, E-DenseNet BC-169 followed by A customized EyeNet. E-DenseNet BC-201-ImageNet ranked the fifth order. In this dataset, it was the first time to notice the big difference between E-DenseNet BC-169 with pre-trained customized weights and the same architecture with the pre-trained ImageNet weights. E-DenseNet BC-169-ImageNet was very near to Inception V3 [44], ResNet50 [40], and VGG19 [45]. On the other hand, the comparison of the calculation time shows that the E-DenseNet models take less time than the other models. E-DenseNet BC-169-ImageNet, E-DenseNet BC-201-ImageNet, and E-DenseNet BC-169 are equal in the calculation time of 4m. E-DenseNet BC-121-ImageNet and E-DenseNet BC-121-ImageNet are also equal. Their calculation time is 2m. On the contrary, the InceptionV3 model takes a long time, about 50m, followed by ResNet50 that takes 33m. After that VGG19 model takes about 28m, and lastly, the customized EyeNet model takes 15m.

Table 9 shows the comparison between the nine models on the IDRiD dataset. We can notice that the proposed E-DenseNet BC-121 architecture model achieved higher ACC, SEN, SPE, DSC, and QKS than other models. It achieved 93%, 96.7%, 72%, 96%, and 0.94 respectively. E-DenseNet BC-169-ImageNet came in the second order, then E-DenseNet BC-121-ImageNet. E-DenseNet BC-201-ImageNet and E-DenseNet BC-169 were in the fourth and fifth ranks. The customized EyeNet model came in the sixth order with a difference of 20%, 10.2%, and 0.26 in ACC, DSC, and QKS, respectively. The difference was very high between the proposed E-denseNet BC-121 and the customized EyeNet. This proved that it was very necessary to customize the traditional EyeNet, but it was not enough to utilize it in the prediction. Therefore, it was good to make a hybrid model from the customized EyeNet and the DenseNet BC architecture with 121 depth by using the pre-trained customized weights. The SPE of this dataset was not good enough, but we noticed the big difference between the proposed model and the other models when we made the comparison. In this respect, we observed that the VGG19 [45] came in the seventh order, followed by the InceptionV3 [44]. Finally, we noticed that ResNet50 [40] model came in the last order as it gave the worst results. For the calculation time, InceptionV3, ResNet50, VGG19, and the customized EyeNet models take 27.5m, 23.5m, 17.05m, and 16.4m, respectively. On the other hand, the E-DenseNet models take 7m, 6m, 4m, 3m, and 3m for E-DenseNet BC-201-ImageNet, E-DenseNet BC-169-ImageNet, E-DenseNet BC-169, E-DenseNet BC-121-ImageNet, and E-DenseNet BC-121, respectively.

**Table 10:** CM on the APTOS 2019 dataset

	Normal	Mild	Moderate	Severe NPDR	PDR
Normal	319	13	11	10	8
Mild	6	45	18	1	4
Moderate	6	14	165	10	5
Severe NPDR	0	1	3	35	0
PDR	2	1	1	0	55

From Table 10, we can notice that the normal images that correctly predicted are 319 images, which equal about 88.4%. The mild cases that are correctly predicted are 45 images, which equal about 60.8%. The moderate cases that are correctly predicted are 165 images, which equal about 82.5%. The severe NPDR cases that are correctly predicted are 35 images, which equal about 89.7%. Finally, the PDR cases that are correctly predicted are 55 images, which equal about 93.2%.

**Table 11:** CM on the EyePACS dataset

	Normal	Mild	Moderate	Severe NPDR	PDR
Normal	272	1	1	0	0
Mild	10	170	0	4	0
Moderate	4	3	157	0	0
Severe NPDR	0	0	0	192	0
PDR	2	8	6	0	170

From Table 11, we can notice that the normal images that correctly predicted are 272 images that equal about 99.2%. The mild cases that are correctly predicted are 170 images that equal about 92.3%. The moderate cases that are correctly predicted are 157 images that equal about 95.7%. The severe NPDR cases that are correctly predicted are 192 images that equal 100%. Finally, the PDR cases that are correctly predicted are 170 images that equal about 91.3%.

**Table 12:** CM on the MESSIDOR dataset

	Normal	Mild	Moderate	PDR
Normal	6	1	0	0
mild	0	2	1	0
moderate	0	0	4	0
Severe NPDR	0	0	0	6

From Table 12, we can notice that the normal images that correctly predicted in MESSIDOR dataset are 6 images that equal about 85.7%. The mild cases that are correctly predicted are two images that equal about 66.7%. The moderate cases that are correctly predicted are four images that equal about 100%. The severe NPDR cases that are correctly predicted are six images that equal 100%.

**Table 13:** CM on the IDRiD dataset

	Normal	Mild	Moderate	Severe NPDR	PDR
Normal	27	0	0	0	0
Mild	1	3	0	0	0
Moderate	2	0	24	1	0
Severe NPDR	0	0	1	14	0
PDR	0	0	1	0	9

From Table 11, we can notice that the normal images that correctly predicted are 27 images that equal 100%. The mild cases that are correctly predicted are 3 images that equal about 75%. The moderate cases that are correctly predicted are 24 images that equal about 88.8%. The severe NPDR cases that are correctly predicted are 14 images that equal 93.3%. Finally, the PDR cases that are correctly predicted are 9 images that equal about 90%.

## Discussion

In this section, we provide a comparison between the proposed E-DenseNet system and other methods that are conducted in the literature. The proposed system achieved the best results compared to the others. We observed that our proposed system outperforms VGG16, VGG19 [45], ResNet50 [40], Inception V3 [44], and the traditional EyeNet [16]. When we customized the traditional EyeNet by optimizing the hyperparameters and layers, we found that the results need to be increased to achieve the DR grades’ satisfied predictions. So, we built the hybrid model to save memory and time from one side and benefit small datasets from the other side (Table 13).

In 2016, Doshi et al. [46] achieved 0.386 for QKS on the EyePACS dataset, which is less than ours by 0.6 on the same dataset. In the year 2017, Wang and Yang [47] achieved 0.85 for QKS. In 2018, Pan et al. [48] achieved 78.4% ACC on EyePACS by ResNet18 to detect DR grades, but our proposed system achieved 96.8%, 98.3%, and 0.97 for ACC, DSC, and QKS. On the other hand, in the year 2018, Islam et al. [19] achieved 85.1% for QKS, which is less than ours by 12%. In 2019, Hagos and Kant [23] achieved 90.9% for ACC. They classified only two classes(healthy/unhealthy) cases on 2500 colored fundus images of the KAGGLE dataset without using data augmentation. Khalifa et al. [22] achieved 97.7% for training ACC on APTOS 2019 dataset using DenseNet. Still, they achieved 99.4% for training ACC and 84% for validation ACC, and 81.7% for testing ACC on the same dataset. We achieved 0.75 for QKS. In the year 2020, the challenges are increased. A lot of literature, such as Shah et al. [27] achieved 0.95 for kappa, but it is achieved only for the DR severity detection on the MESSIDOR dataset. Patil et al. [25] achieved 89.1% for ACC. Vora and Shrestha [49] achieved 76% for average ACC in binary classification to detect only the presence/absence of DR without diagnosing the various DR grades on EyePACS. Tymchenko et al. [28] achieved 99% for SEN and SPE, and 0.92 for QKS for binary classification on APTOS 2019 dataset. Gadekallu et al. [50] achieved 97% for ACC in DR binary classification. The authors concluded that their method might not give the same performance implemented on the low dimensional datasets as it may fall into overfitting. In this respect, we put utilized data augmentation. Finally, in the year 2021, Aswathi et al. [30] achieved 78% for ACC to detect DR grades on MESSIDOR dataset. We achieved 91.6% for ACC on the same dataset. Amalia et al.[51] achieved 90% for ACC on the MESSIDOR dataset by combining the CNN and long short-term memory (LSTM). The authors performed binary classification, while our system provides diagnosing of the healthy and four DR grades.

**Table 14:** The proposed system results on the four datasets due to ACC, SEN, SPE, DSC, QKS, and T(m) performance measures

Dataset	ACC %	SEN %	SPE %	DSC %	QKS	T (m)
IDRiD	93	96.7	72	96	0.94	3
MESSIDOR	91.6	95	58	95.1	0.91	2
EyePACS	96.8	98.3	72	98.3	0.97	5
APTOS 2019	84	94	74	87	0.8	4
Average	91.35	96	69	93.3	0.90	3.5

**Fig. 10:**
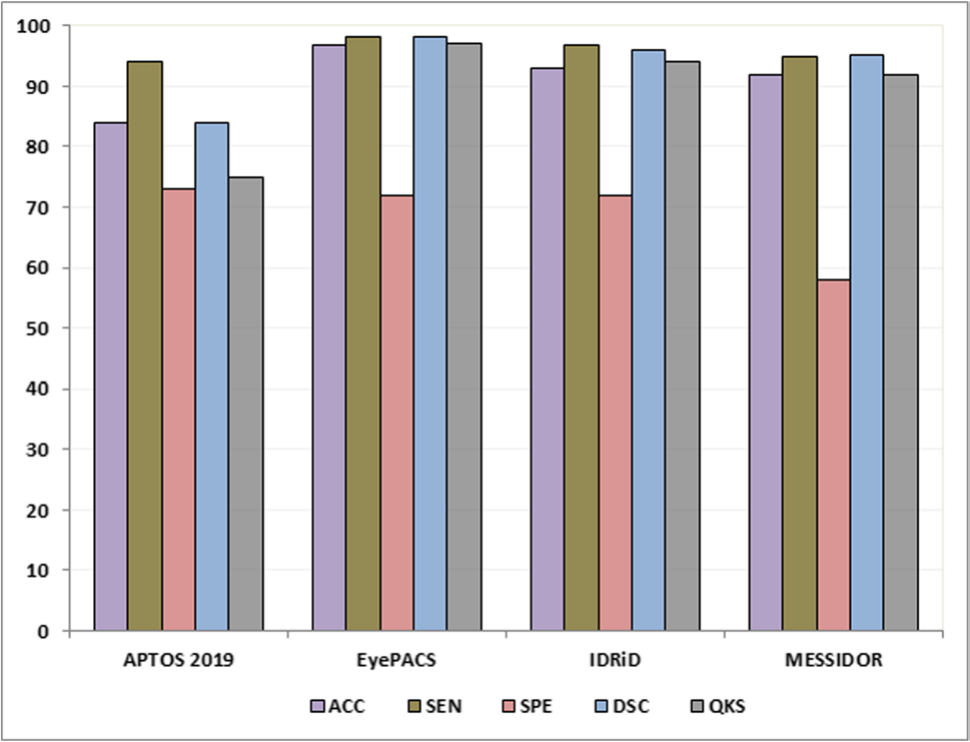
The averages of ACC, SEN, SPE, DSC, and QKS of the proposed CAD system on the four benchmark datasets APTOS 2019, EyePACS, IDRiD, and MESSIDOR

**Fig. 11:**
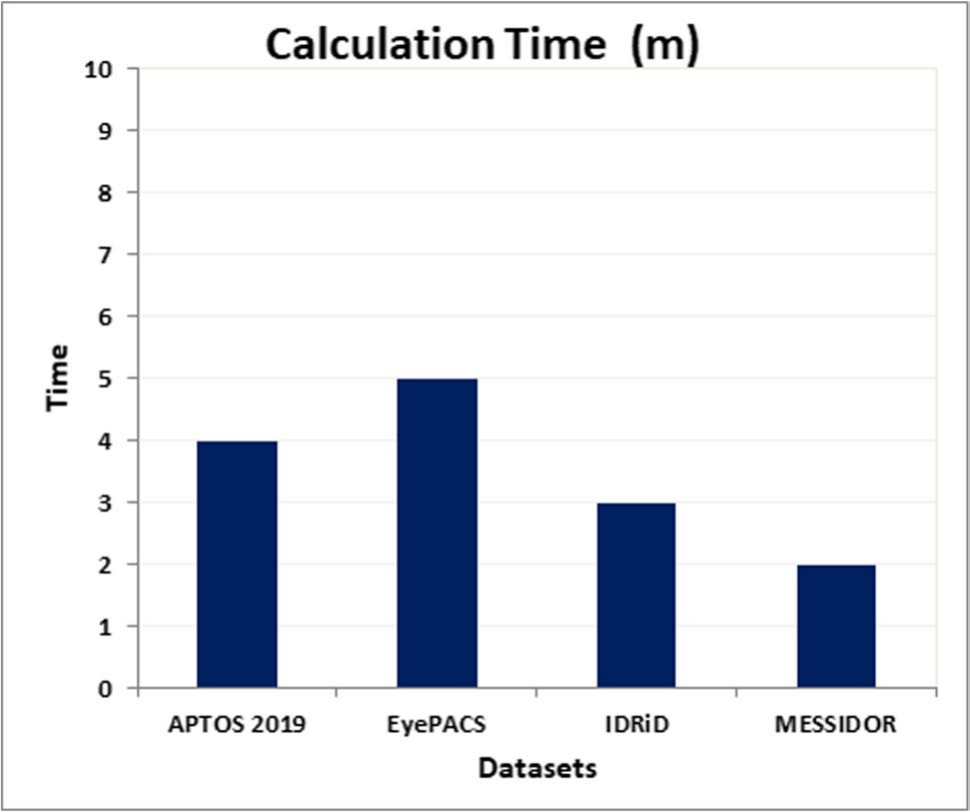
The averages of calculation time (T) in minutes (m) of the proposed CAD system on the four benchmark datasets APTOS 2019, EyePACS, IDRiD, and MESSIDOR

From Table 14, we can observe that the proposed system based on E-DenseNet model achieved 91.35%, 96%, 69%, 93.3%, 0.90, 3.5m for averages of ACC, SEN, SPE, DSC, QKS, and T(m), respectively. On the other hand, Fig. 10 represents the average results of applying the proposed system on the four datasets (IDRiD, MESSIDOR, EyePACS, and APTOS 2019). Figure 11 represents the average results of applying the proposed system on the aforementioned datasets due to the calculation time. The average calculation time is 3.5m.

From Table 14, we can observe that SPE is lower than SEN in all datasets that is returns to some causes such as:

- The similarity of classes. Most DR lesions (EX, MA, HM, VB, CWS, NV, and others) take the same color, shape, and other features of the fundamental human eye contents (OD, fovea, and BV).
- The illuminations, poor quality, light shadow, noise, blurring, focusing, and exposure, and artifacts may be viewed as abnormal signs in the color fundus image. These factors affect the training procedures and the model performance. Therefore, we utilized the preprocessing processes to reduce the influence.
- There are many features of the color fundus images, while the difference between these features is very few.
- When wanting to train the model with very high-resolution fundus images, for small lesions to be easier detected accurately. However, the computational complexity, as well as the (vanishing and exploding) gradient problem of CNNs, prevents this.
- The correct classification of the mild DR cases depends on extracting subtle features from these high-resolution images. The misclassification was more common for mild DR than the other classes. The details of mild cases are harder to identify because their size and number are very small and little (about 1% of the image).
- Dataset imbalance.

From the previous demonstration, there are some misclassified images because of the camera malfunction. For example, in the EyePACS dataset, total black images are 1050, 1475, 1557, 10194, 10698, and 10924 for left and right eyes. White lines are found in 1061, 1499, 10029, and 10567 for left and right eyes. High black shadow is found in images number 10131 for the left eye. A big orange blot area is found in image number 10440 for the left eye. Image number 10147 for the left eye is predicted as FP. In the MESSIDOR dataset, image number 58065 is covered by shadow. Therefore, it is predicted as severe while it is normal. In addition, image number 61804 is normal but is detected as moderate because of the strong yellow spots that are similar to hard EX. For the same reasons, IDRiD_010 image is moderate and detected as PDR. In APTOS 2019, image number 002c21358ce6 is normal and predicted as severe NPDR.

In experiments, we tried to start by the three CONV layers of the traditional EyeNet model before DenseNet, but the model reported less performance. Therefore, we applied the DenseNet to provide us with deeper concatenated features and then extended the extraction by using the three optimized CONV layers of the EyeNet model.

The advantages of the proposed CAD system based on E-DenseNet are as follows. First, it has a comprehensive style to diagnose the various DR grades. Second, it is more accurate than others. Third, it can be applied to different real small/large ML datasets with different settings. Fourth, it saves time, memory, and effort by using the pre-trained DenseNet model and DL rather than hand-crafted techniques. Finally, it is one of the few studies conducted on the DR grading field. In contrast, most of the conducted research in analyzing DR disease only detects the presence/absence of DR or segment its lesions [5]. This approach’s limitations are that the accuracy needs to be increased somewhat, and the AUC value of the normal class is only up to 35% except in the MESSIDOR dataset, which AUC of normal cases is 67%.

## Conclusion

DR is a very progressive disease, which if not detected early will result in blindness suddenly. Therefore, continuous auditing and screening are needed. But, fundus images are like other medical scans. They suffer from noise, artifacts, low contrast, and poor quality. Besides, they have few differences between their features, which leads to hard differentiation between the different characteristics. Moreover, the variety of the lesions that accumulate the DR grades. Thus, the hand-crafted methods to diagnose the different DR grades burden the developer. In contrast, the deep learning techniques solve feature extraction problems, such as CNN models that achieve high success in multi-label classification problem-solving. We integrated two deep learning CNN models, EyeNet and DenseNet models, to produce the E-DenseNet model to accurately diagnose the healthy and DR cases from different color fundus images from four different benchmark datasets. In the future, we want to contribute new ideas and focus on applying the proposed system to other imaging modalities, such as OCTA. These imaging modalities can collect different diseases features simultaneously, such as DR, glaucoma, and age-related macular degeneration.

## Declarations

This research study was conducted retrospectively using human subject data made available in open access by: –   https://idrid.grand-challenge.org/ –   http://www.adcis.net/en/third-party/messidor/ –   https://www.kaggle.com/c/aptos2019-blindness-detection –   https://www.kaggle.com/beatrizsimoes/eyepacs
